# Mizoribine provides effective treatment of sequential histological change of arteritis and reduction of inflammatory cytokines and chemokines in an animal model of Kawasaki disease

**DOI:** 10.1186/1546-0096-9-30

**Published:** 2011-09-29

**Authors:** Kei Takahashi, Toshiaki Oharaseki, Tomokazu Nagao, Yuki Yokouchi, Hitomi Yamada, Noriko Nagi-Miura, Naohito Ohno, Tsutomu Saji, Tomio Okazaki, Kazuo Suzuki

**Affiliations:** 1 Department of Pathology, Toho University Ohashi Medical Center, Meguro-ku, Tokyo, 153-8515, Japan; 2 Inflammation Program, Dept. of Immunology, Chiba University Graduate School of Medicine, Chuo-ku, Chiba, 260-8670, Japan; 3 Laboratory for Immunopharmacology of Microbial Products, School of Pharmacy, Tokyo University of Pharmacy and Life Science, Hachioji, Tokyo 192-0392, Japan; 4 Department of Pediatrics, Toho University Omori Medical Center, Ota-ku, Tokyo, 143-8541, Japan; 5 Kure Kyosai Hospital, Kure, Hiroshima, 737-8505, Japan

**Keywords:** Kawasaki disease, an animal model, IVIg, coronary arteritis, inflammatory cytokines and chemokines, mizoribine

## Abstract

**Background:**

Intravenous immunoglobulin (IVIg) treatment results in an effective response from patients with acute-phase Kawasaki disease (KD), but 16.5% of them remain nonresponsive to IVIg. To address this therapeutic challenge, we tried a new therapeutic drug, mizoribine (MZR), in a mouse model of KD, which we have established using injections of *Candida albicans *water-soluble fractions (CAWS).

**Methods:**

CAWS (4 mg/mouse) were injected intraperitoneally into C57BL/6N mice for 5 consecutive days. MZR or IgG was administered for 5 days. After 4 weeks, the mice were sacrificed and autopsied, the hearts were fixed in 10% neutral formalin, and plasma was taken to measure cytokines and chemokines using the Bio-Plex system.

The incidence of panvasculitis in the coronary arteries and aortic root was 100% in the control group. The incidence of panvasculitis in the MZR group decreased to 50%. Moreover, the scope and severity of the inflammation of those sites were significantly reduced in the MZR group as well as the IgG group. On the other hand, increased cytokines and chemokines, such as IL-1α, TNF-α, KC, MIP-1α, GM-CSF, and IL-13, in the nontreatment group were significantly suppressed by treatment with MZR, but the MCP-1 level increased. In addition, IL-1α, TNF-α, IL-10, IL-13, and MIP-1α were suppressed by treatment in the IgG group.

**Results:**

The incidence of panvasculitis in the coronary arteries and aortic root was 100% in the control group. The incidence of panvasculitis in the MZR group decreased to 50%. Moreover, the scope and severity of the inflammation of those sites were significantly reduced in the MZR group as well as the IgG group. On the other hand, increased cytokines and chemokines, such as IL-1α TNF-α, KC, MIP-1α, GM-CSF, and IL-13, in the nontreatment group were significantly suppressed by treatment with MZR, but the MCP-1 level increased. In addition, IL-1α, TNF-α, IL-10, IL-13, and MIP-1α were suppressed by treatment in the IgG group.

**Conclusion:**

MZR treatment suppressed not only the incidence, range, and degree of vasculitis, but also inflammatory cytokines and chemokines in the plasma of the KD vasculitis model mice, suggesting that MZR may be useful for treatment of KD.

## Background

Kawasaki disease (KD) is an acute febrile illness that manifests mainly in infancy and early childhood [1]. The most important complication of KD is coronary arteritis, which leads to formation of aneurysms. KD has attracted special interest because it may cause ischemic heart disease in children due to thrombosed coronary aneurysms [2]. Since the etiology and development of KD are thought to be due to the dysfunction of the immune system, intravenous immunoglobulin (IVIg) during the early acute phase has been used with an excellent response in most patients [3]. However, 16.5% of patients did not respond to the first IVIg treatment [4], and some nonresponders to the first IVIg treatment manifested severe coronary arteritis with large aneurysm [5]. Therefore, additional treatments have been tried on the nonresponders to the first treatment with IVIg. To date, a second IVIg treatment [6], plasmapheresis [7-10], pulse steroids [11], cyclophosphamide plus steroids [12], ulinastatin as an elastase inhibitor [13-16], cyclosporin A plus steroids and methotrexate plus steroids [17,18], and anti-tumor necrosis factor-α (infliximab) therapy [19-23] have been tried. Thus, for treatment of patients with KD who do not respond to IVIg, other medicines for immune response and suppression of lymphocyte proliferation have been applied due to immune dysfunction in the patients. One immune modulating medicine, mizoribine (MZR), a drug that inhibits synthesis of purine compounds (GMP), blocks proliferation of lymphocytes and will be useful for application to nonresponders to IVIg treatment. MZR has long been used as therapy for kidney transplantation, lupus nephritis, nephrotic syndrome, and rheumatoid disease with few side-effects [24]. Moreover, it has been reported to have been used for lupus nephritis, nephrotic syndrome, and IgA nephritis in children [25-28], and as a maintenance therapy in anti-neutrophil cytoplasmic autoantibody (ANCA)-associated renal failure, frequently relapsing nephrotic syndrome, and purpura nephritis [29,30]. Therefore, MZR will be a valuable therapeutic strategy for patients with KD who are nonresponsive to IVIg.

Prior to a clinical trial in children with KD, it was necessary to test MZR in a mouse model of KD, which has been established. The model we chose was the mouse model in which coronary arteritis can be induced by administration of *Candida albicans *water-soluble fractions (CAWS) [31]. This model mouse has previously been useful for evaluation of other drug treatments.

Therefore, in the present study, we tested MZR as a immunomodular for treatment of this CAWS-induced coronary arteritis. The evaluation of MZR was performed by histopathological findings and profiles of chemokines and cytokines. Also, this treatment effect was compared with that of IgG.

## Methods

### Animals

Four-week-old male C57BL/6N mice were purchased from Charles River Japan (Yokohama, Japan). All mice were kept under specific pathogen-free (SPF) conditions, according to the guidelines for animal care of the National Institute of Infectious Diseases in Tokyo (NIID).

### Preparation of CAWS

CAWS was prepared from *C. albicans *strain IFO1385 in accordance with the reported method [31]. Briefly, 5 liters of medium (C-limiting medium) was added to a glass incubator, and the culture was maintained for 2 days at 27°C while air was supplied at a rate of 5 liters/min and the mixture was swirled at 400 rpm. Following culture, an equal volume of ethanol was added. After allowing this to stand overnight, the precipitate was collected. After dissolving the precipitate in 250 ml of distilled water, ethanol was added and the mixture was allowed to stand overnight. The precipitate was collected and dried with acetone to obtain CAWS.

### Administration of MZR and IgG to the mice

CAWS (4 mg/mouse/day) in a volume of 0.2 ml was intraperitoneally injected into a C57BL/6N mouse (4-week old male) on each of 5 consecutive days. Subsequently, MZR (a kind gift of Asahikasei Pharma Corporation (Tokyo, Japan)) was administered at a dose of 30 mg/kg/day intraperitoneally for 5 days from the third day of CAWS injection (MZB group), according to the schecule for treatments such as IgG for patients with KD, and the dosage as described elsewhere [32]. Mice for the control group were intraperitoneally treated with 0.2 ml of Dulbecco's phosphate-buffered saline (PBS). After 35 days, the mice were sacrificed by carbon dioxide asphyxiation; autopsy was performed to obtain plasma, and hearts were fixed with 10% neutralized formalin. For a positive control, treatment with intraperitoneal human IgG (Kenketsu Glovenin I, a kind gift of Nihon Pharmaceutical Co. Ltd., Tokyo, Japan) was performed at a dose of 400 mg/mouse/day, or for a negative control saline containing 0.1% glucose (SG) was injected for 5 days according to the same procedures as described elsewhere (IgG group) [33]. The start date of the drug administration was based on the results that the administration from the third experimental day had been the most effective to suppress the development of vasculitis.

### Histological evaluation

The fixed hearts were embedded in paraffin and sectioned. To observe the histological changes in the coronary arteries and the aorta in detail, 20 to 30 horizontal step sections per mouse were made every 20 μm. Hematoxylin and eosin (H&E)-stained sections were prepared by using routine techniques for examination by light microscopy [31]. First, we investigated the incidence of mice with panvasculitis in each group. Panvasculitis was defined as inflammation of all layers of the walls of the coronary arteries and/or the aorta. Then, for quantitative evaluation of vascular inflammation, we divided the area of the aortic root and coronary arteries into five segments and graded the intensity of inflammation in each segment as follows: score 3, panvasculitis; score 2, inflammation involving the tunica intima and adventitia; score 1, inflammation localized to the tunica intima; and score 0, no inflammatory cell infiltration in the vascular wall. A section with the severe inflammation was observed in each segment. The scope of inflammation was defined as the number of segments evaluated as score 1 or more in each mouse, and the severity of the arteritis was defined as the average score of the five segments in each mouse.

### Measurement of cytokines and chemokines with Bio-Plex

Cytokines and chemokines in the plasma of mice autopsied were measured by a Bio-Plex system. An aliquot of serum (12 μl) collected from peripheral blood and diluted 4-fold with the dilution solution was measured for concentration of cytokines by the 23-Plex kit using Bio-Plex 200 according to the manufacturer's protocol and analyzed by the Bio-Plex Luminex 100 XYP instrument (Bio-Rad, Hercules, California, USA). We assayed the following 23 cytokines and chemokines: IL-1α, IL-1β, IL-2, IL-3, IL-4, IL-5, IL-6, IL-9, IL-10, IL-12p40, IL-12p70, IL-13, IL-17, eotaxin, G-CSF, GM-CSF, INF-γ, KC, MCP-1, MIP-1α, MIP-1β, RANTES, and TNF-α as estimated with a single assay to a single standard curve described in the kit instructions. Concentrations of cytokines and chemokines were calculated using Bio-Plex Manager 3.0 software (Bio-Rad, Tokyo) with a five-parameter curve-fitting algorithm applied for standard curve calculations [34].

### Statistical analysis

Fisher's exact probability test was used to analyze the differences in the incidence of arteritis among the groups. The data on the scope and severity of the arteritis and cytokine/chemokine levels were analyzed using the two-sample *t*-test. A value of *P *< 0.05 was considered statistically significant.

## Results

### Histological evaluation of panvasculitis in treatment with MZR

**Figure 1:**
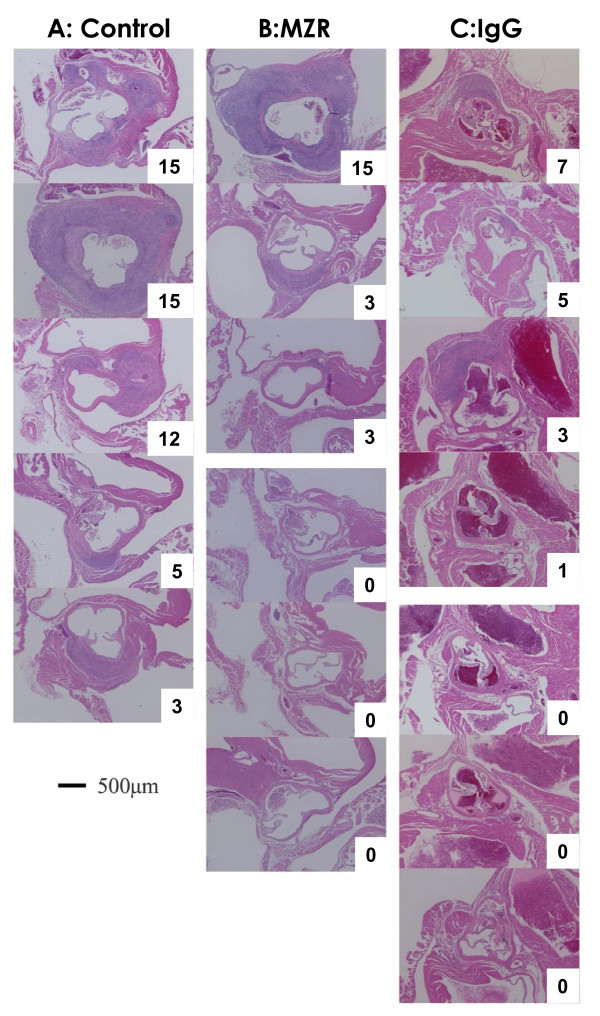
**Histological observations of coronary arteritis induced by treatment with MZR and IgG**. A, Control (PBS); B, MZR group; C, IgG group. Each micrograph represents an individual mouse. H&E stain, Bar: 500 μm. Numbers (white) are coronary arteritis score.

Panvasculitis developed in the coronary arteries and the aortic root, and histology was similar to that previously described [33]. Specifically, vascular changes were classified as proliferative inflammation that consisted mainly of large mononuclear cells such as histiocytes and fibroblasts and of a small number of neutrophils. The normal structure of the arteries was completely destroyed, and the internal elastic lamina, external elastic lamina, and smooth muscle layer of the tunica media were severely damaged. However, fibrinoid necrosis was not observed in any of the mice. In addition, the histology of panvasculitis was similar in the three groups (Figure 1).

### Decrease of coronary arteritis by treatment with MZR

**Figure 2:**
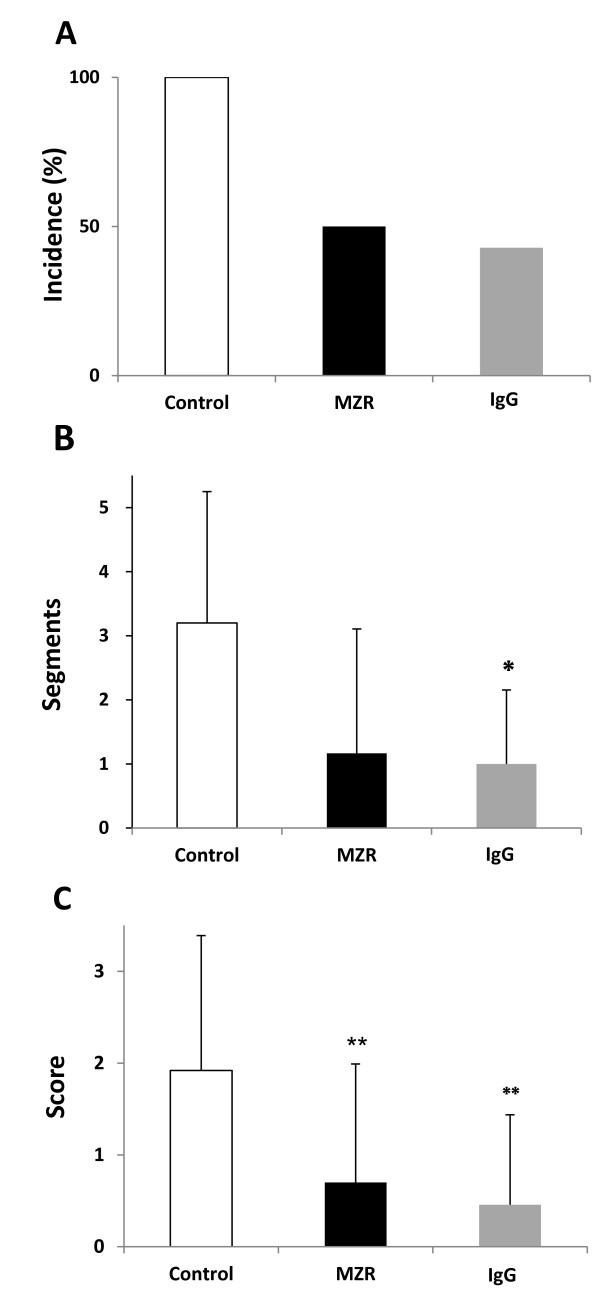
**Decrease of incidence of panvasculitis, segment score, and severity score of coronary arteritis by treatment with MZR and IgG**. A, incidence of development of panvasculitis; B, scope as number of segments with inflammation evaluated as score 1 or more at aortic root and coronary arteries; C, severity score of each segment. Data are expressed as mean ± SD of results from three individuals. *P < 0.05 and **P < 0.01 (Student's *t*-test).

Panvasculitis of the coronary arteries and the aortic root was observed in 5 of 5 mice (100%) in the nontreated control group. On the other hand, the incidence of panvasculitis in the MZR group was 3 of 6 mice (50%), and the IgG group as an effective control showed 3 of 7 (43%) (Figure 2A). In addition, the number of segments evaluated as score 1 or more in each MZR group was decreased compared with the nontreated control group (*P *= 0.06), and the scope of inflammation in IgG groups was significantly lower than in the control group (*P *< 0.05) (Figure 2B). Furthermore, the severity of the arteritis, i.e., the scores of each of five segments in the mice in the MZR and IgG groups, was significantly lower than in the nontreated control group (*P *< 0.01) (Figure 2C).

### Reduction of inflammatory cytokines and chemokines by treatment with MZR and IgG

**Figure 3:**
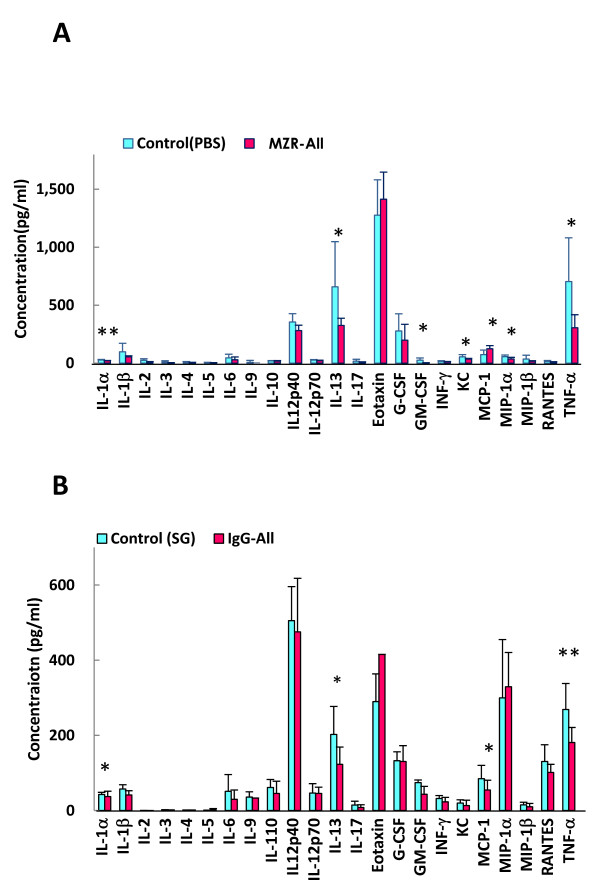
**Reduction and enhancement of cytokines and chemokines by treatment with MZR and IgG**. A, MZR treatment; B, IgG treatment. SG: saline including 0.1% glucose. Data are expressed as mean ± SD of results from three individuals. *P < 0.05 and **P < 0.01.

Inflammatory cytokines IL-1α, TNF-α, chemokines KC, MIP-1α, GM-CSF, and Th2, and cytokine IL-13 in plasma of mice, which were inoculated with CAWS in the control group, were elevated (Figure 3). However, in the MZR group, plasma levels of inflammatory cytokines IL-1α (*P *< 0.01) and TNF-α (*P *< 0.05), and chemokines KC (*P *< 0.01), MIP-1α (*P *< 0.01), and GM-CSF (*P *< 0.05) were significantly suppressed (Figure 3A). Inversely, the MCP-1 level increased with MZR treatment (Figure 3A). On the other hand, IL-1α (*P *< 0.05), TNF-α (*P *< 0.05), IL-10 (*P *< 0.05), and IL-13 (*P *< 0.01) were suppressed by administration of IgG (Figure 3B).

**Figure 4:**
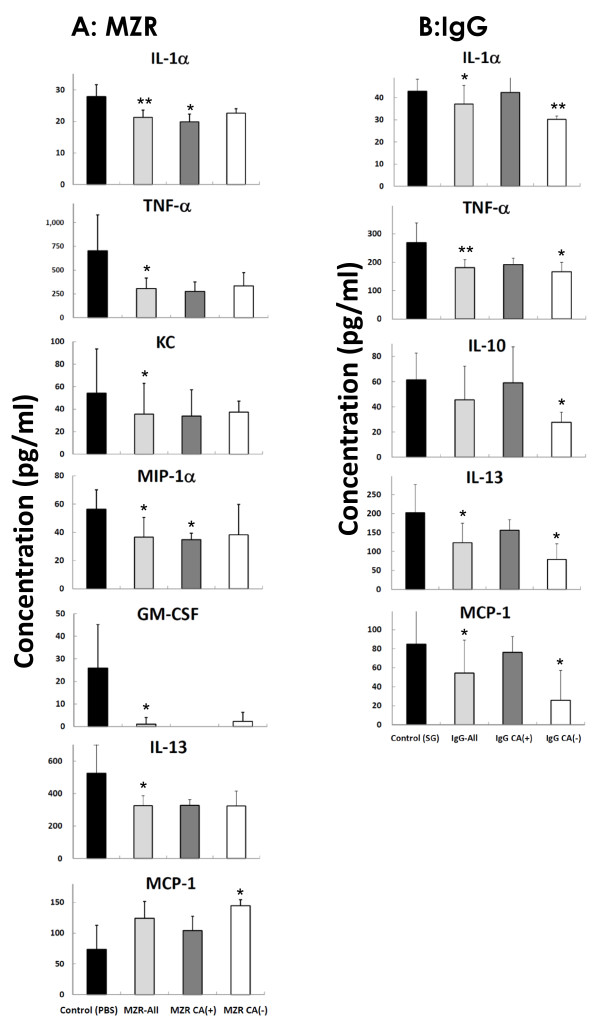
**Reduction and enhancement of cytokines and chemokines in coronary arteritis treated with MZR and IgG**. A, MZR treatment; B, IgG treatment. CA(+): coronary arteritis score > 0, CA(-): coronary arteritis score = 0, SG: saline including 0.1% glucose. Data are expressed as mean ± SD of results from three individuals. *P < 0.05 and **P < 0.01.

Furthermore, we analyzed levels of cytokines/chemokines in plasma, which were related with suppression of the development of coronary arteritis by treatment with MZR. As shown in Figure 4A, the suppression levels were almost the same in all plasmas of MZR-treated mice. These results are not the same as those in the IgG group, showing good response for suppression of the development of coronary arteritis (Figure 4B).

## Discussion

### Decrease of coronary arteritis by treatment with MZR

We here have shown the efficacy of MZR on vascular inflammation by using a KD vasculitis mouse model to develop alternative treatments for KD patients who are nonresponsive to IVIg treatment. The results here show that the incidence, scope, and degree of inflammation of the coronary arteries and the aortic root were suppressed by MZR administration. Coronary arteritis in this CAWS-induced vasculitis mouse model is also suppressed after administration of IVIg [33]. Furthermore, we have also demonstrated that the anti-TNF-α therapy that has been shown to be effective in treating some children unresponsive to IVIg therapy also dramatically suppresses the development of vasculitis in this mouse model of KD (manuscript in preparation). Thus this mouse model appears to be valuable for evaluation of alternative therapies for KD arteritis.

### Reduction of inflammatory cytokines and chemokines by treatment with MZR

Some cytokines and chemokines such as IL-1β, IL-2, sIL-2R, IL-4, IL-6, IL-8, IL-10, IL-12, IL-15, RANTES, MCP-1, M-CSF, G-CSF, and MIPs are elevated in the blood of patients with acute-phase KD. Some elevated cytokines and chemokines are decreased by IVIg treatment in the acute phase, when it is effective [35]. On the other hand, IL-6, TNF-α, IL-4, and IL-12 were increased in the plasma of the KD mouse model induced with *C. albicans*-derived substances (CADS) [36]. Moreover, IL-6 and IFN-γ in splenocytes administered CAWS in C57BL/6 mice were elevated [37]. With IVIg treatment of KD model mice induced with CAWS, elevated proinflammatory cytokines IL-1α, TNF-α, IL-10, IL-13, and MCP-1 were decreased in our data. Furthermore, chemokines IL-1α, TNF-α, KC, GM-CSF, IL-13, and MIP-1α in plasma of autopsied mice were decreased in the MZR treatment group in the present study. The results with MZR treatement show similar effects as well as IgG treatment for KD model mice. However, supression levels of IL-1α, TNF-α, IL-10, IL-13, and MIP-1α in the recovery group from the coronary arteritis (CA(-)) in the MZR group differed from those in the IgG group. Levels in the recovery group (CA(-)) after MZR treatment were not suppressed, whereas those in the CA(-) group after IgG treatment were suppressed in the present study, which suggests that MZR may have a stronger effect than a high dose of IgG (400 mg/kg/day for 5 days). Because these cytokines/chemokines decrease slightly after MZR treatment, they may have a role in the development of coronary arteritis in the KD model.

### Effective treatment with MZR of model mice for KD induced by CAWS

In the present study, MZR treatment of the KD model mice significantly suppressed the development of coronary arteritis associated with significant suppression of levels of proinflamatory cytokines and chemokines in plasma. These results suggest association of the suppression of lymphocyte proliferation with MZR [38]. The mode of action of MZR is that it mainly blocks immunosuppression related to lymphocyte proliferation through inhibition of purine synthesis [32,39,40]. In the present study, the incidence of panarteritis decreased to half, and both the scope and severity of inflammation were limited after administration of MZR. In addition to the lymphocyte action, these observations suggest that MZR may act on functions of monocytes/macrophages and neutrophils, which are mainly involved in the development of inflammation, resulting in the possible suppression of coronary arteritis through suppression of proinflammatory cytokines and chemokines released from these cells. Indeed, recently, MZR acted to inhibit functions of lymphocytes as well as those of macrophages, such as migration and production of Nitrous Oxide Systems (NOS), IL-1β, and TNF-α in a dose-dependent manner [41,42]. Furthermore, in the mixed lymphocyte reaction method (MLR) of human peripheral blood mononuclear cells, the IC_50 _is 1 μg/ml [43]. In addition, MLR of T-cells in human peripheral blood, which are stimulated with anti-CD3 monoclonal antibody, shows an IC_50 _of less than 1 μg/ml for MZR and also phorbol myristate stimulation less than 5 μg/ml [44]. In addition, MZR also inhibits activation of M1 macrophages [42], which are classified as inflammatory, showing tissue injury and activation with IFN-γ. In the present study, suppression profiles of proinflammatory cytokines and chemokines by MZR treatment of KD model mice seem to be associated in the literature with those in the M1 macrophage. Therefore, the effect of MZR on KD model mice may be to inhibit the proliferation of lymphocytes and activation of macrophages and neutrophils associated with elevation of proinflamatory cytokines and chemokines.

Based on these observations, suppression of development of coronary arteritis associated with suppression of proinflammatory cytokines and chemokines by MZR treatment for the KD model mice suggests that MZR may be useful for patients with KD in the acute phase. MZR has been used as therapy for kidney transplantation, lupus nephritis, nephrotic syndrome, and rheumatoid disease with few side effects [24]. Furthermore, MZR has been used as maintenance treatment for ANCA-associated vasculitis, frequently relapsing nephrotic syndrome, and purpura nephritis [29,30]. Clinical use will be recommended for immune dysfunctions when the safety of long-time use becomes known. Therefore, MZR is a possible therapy for patients with KD who are nonresponsive to IVIg.

## Conclusions

MZR treatment suppressed not only the incidence, range, and degree of vasculitis, but also inflammatory cytokines and chemokines in the plasma of the KD vasculitis model mice. It appears likely that MZR may prove to be a useful for alternative treatment for KD.

## Abbreviations used

ANCA: anti-neutrophil cytoplasmic autoantibody; CAWS: *Candida albicans *water-soluble fractions; H&E: hematoxylin and eosin; IVIg: intravenous immunoglobulin; KD: Kawasaki disease; MLR: mixed lymphocytes reaction method; MZR: mizoribine; NOS: Nitrous Oxide Systems: PBS; Dulbecco's phosphate- buffered saline; SG: saline containing 0.1% glucose.

## Competing interests

The authors declare that they have no competing interests.

## Contribution of authors

KT: Histological evaluations of coronary arteritis. TO: Histological evaluations of coronary arteritis. TN: Measurement and analysis of cytokines and chemokines. YY: Measurement and analysis of cytokines and chemokines. HY: Histological evaluations of coronary arteritis. NNM: Preparation of CAWS. NO: Preparation of CAWS. TS: Planning treatments with MZR and IgG, and clinical evaluation. TO: Planning treatments with MZR and IgG, and clinical evaluation. KS: Measurement and analysis of cytokines and chemokines, correspondence to all evaluation of this study. All authors read and approved the final manuscript.
